# One-Pot Dual-Wavelength 3D Printing Breaks Free from Support Constraints

**DOI:** 10.1021/acscentsci.5c00958

**Published:** 2025-06-09

**Authors:** Jared Cason Head, Syed Muhammad Usama

**Affiliations:** † Department of Chemistry, 12346 University of Texas at San Antonio, 1 UTSA Circle, San Antonio, Texas 78249, United States

## Abstract

The Page and Huang groups employ orthogonal light sources to fabricate freestanding 3D structures that eliminate the need for traditional support material.

## Graphical abstract

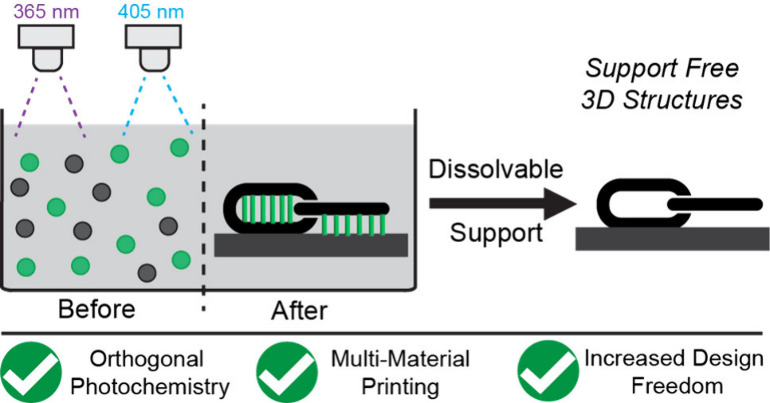

Additive manufacturing (AM) via vat photopolymerization (VPP)—particularly techniques like digital light processing (DLP)—enables the rapid fabrication of polymer structures with high spatial resolution. VPP has advanced into an indispensable 3D printing tool for producing multimaterial components for diverse applications. However, limitations arising from the fabrication of intricate structures have posed a large obstacle for its mass adoption.

In two recent studies published in *ACS Central Science*, the Page and Huang groups unveil innovations in digital light processing (DLP) showcasing a quiet revolution in 3D printing—one driven not just by faster hardware or finer resolution, but by smart resin chemistry and the nuanced use of light.

Vat photopolymerization has garnered increased attention in 3D printing owing to its high resolution, scalable production, design freedom, and reduced material waste.^1–3^ VPP relies on light exposure to selectively cure liquid resins, or inks, to create complex geometries that can be utilized in metamaterials,^4^ microfluidics,^5^ and biomedical engineering.^6^ However, fabricating fine suspended features such as arches, hooks, bridges, or overhangs remains a key challenge in 3D printing. Without adequate support components, structures demanding freestanding or mobile features are prone to deform during printing, which limits design freedom. Moreover, supports are often removed through manual postprocessing steps that can obscure or damage delicate details, introducing a major bottleneck in the printing process^7^, prompting the need for innovative curing processes. In their current contribution, the Page and Huang groups report distinct yet convergent solutions using dual-wavelength DLP printing (Figure 1) to simultaneously fabricate robust parts and easily removable supports—all from a single resin formulation and without the need for printer modifications or multiple resin vats.

**Figure 1:**
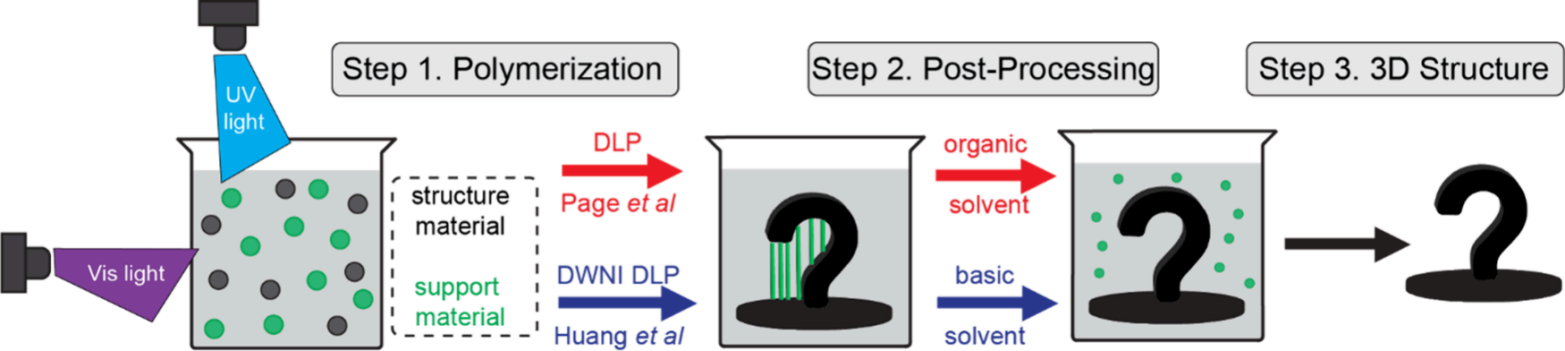
Dual wavelength (UV and Vis) DLP printing from a single resin containing epoxy and acrylate monomers to generate degradable supports along with a built structure. The sacrificial supports are removed postprocessing using either an organic or a basic solvent to generate a free-standing 3D structure. Red and blue arrows signify the differing approaches utilized by the Page and Huang groups, respectively.

The Page group’s strategy^1^ leverages orthogonal photopolymerization mechanisms within a carefully formulated wavelength-selective resin. Under blue or violet light (∼405 nm), acrylate monomers undergo radical chain-transfer polymerization, forming soluble thermoplastics. Simultaneously, UV light (∼365 nm) triggers cationic polymerization of epoxies into cross-linked thermosets, yielding insoluble components that constitute the built structure. Spatially modulated with a DLP projector, this dual-cure chemistry allows for rapid printing (up to 0.75 mm/min) of interlocked joints, unsupported arches, and freestanding chains—all in one build. Postprocessing is refreshingly simple: a 10 min rinse in ethyl acetate at room temperature dissolves the support regions, revealing smooth, structurally resilient features with <5 μm surface roughness and mechanical properties approaching those of commercial plastics (*E* ≈ 1 GPa, σ_m_ ≈ 30 MPa). Layer exposures of just 4–6 s per 50 μm slice enable fine feature resolution (<100 μm), matching or exceeding industry standards.

In a parallel effort, the Huang group^2^ introduced a custom-built dual-wavelength negative imaging (DWNI) DLP printer to achieve similar results but with a different architectural and chemical approach. Their single-resin formulation contains both a carbonate-linked anhydride thermoset network and a UV-curable epoxy system. Visible light selectively cures the degradable anhydride acrylate matrix, while UV light simultaneously cross-links the permanent epoxy regions—all patterned using a single digital micromirror device (DMD). The approach eliminates the complexity of employing dual-vat or dual-projector setups. After printing, a mild thermal postcure enhances the structural network’s fidelity. The sacrificial support material can then be selectively removed in a gentle aqueous base (pH ≈ 11), avoiding harsh solvents and preserving fine interfacial details.

From a chemical standpoint, the two approaches diverge in mechanism yet converge in sophistication. The Page group employs light-orthogonal radical and cationic polymerizations to embed a degradable thermoplastic scaffold within a durable thermoset matrix. In contrast, the Huang group utilizes a dual-cure formulation with anhydride-based support networks that selectively degrade under basic conditions. Both strategies eliminate the need for resin switching by embedding wavelength-selective reactivity directly into a single formulation.

Collectively, both groups showcase how light—not merely as a tool for curing resin, but as a finely tuned sculptor—can drive the next generation of additive manufacturing.

Whether through multicolor resin chemistry or dual-wavelength projection, the future of 3D printing lies in coupling photonics with smart molecular design. By shifting the paradigm from manual postprocessing to automated, chemically programmed support removal, both works move closer toward a vision where complexity comes free of cost—not only in dollars, but also in time, labor, and waste.

These innovations seek to build on a rich history of support strategies. Early techniques involved mechanical breakaway scaffolds^8^ or dissolvable filaments, mostly limited to rigid thermoplastics. Multimaterial resin systems—while promising—have long struggled with cross-contamination, alignment, and inconsistent cure kinetics.^9^ More recently, grayscale photopolymerization and projection-based voxel tuning^10^ hinted at the possibility of printing gradients or hybrid materials, but with limited compositional control. The dual-wavelength methods reported here offer a leap forward in integration and resolution. Sacrificial supports are intentionally programmed to disappear under gentle, selective conditions, opening the door to new applications in microfluidics, soft robotics, and bioprinting, where complex internal channels or delicate overhangs must remain pristine after fabrication.

Looking ahead, dual-wavelength DLP printing opens new frontiers in additive manufacturing. More tunable light sources could enable spatial control over mechanical or optical gradients within a single print. Expanding the resin palette to include conductive, stretchable, or bioresorbable materials would further extend the technology’s reach. From a sustainability perspective, the development of recyclable supports or greener solvents could enhance the environmental case for this approach.

These studies demonstrate that when materials chemistry and optical engineering are skillfully integrated, long-standing limitations in 3D printing can be reimagined—not merely overcome.

Support structures, once seen as a necessary nuisance, are now an intelligent, programmable component of the printing process.

By choreographing photons of different energies, both works dismantle a core constraint of DLP printing: the need for mechanical postprocessing to remove supports. Their distinct strategies—orthogonal multicolor photopolymerization and dual-wavelength base-degradable networks—pave the way toward autonomous, multimaterial manufacturing. In an era demanding faster prototyping, reduced waste, and greater geometric freedom, dual-wavelength DLP methods presented in this context are poised to become a cornerstone of next-generation additive manufacturing.

## References

[1] Mason K. S., Kim J.-W., Recker E. A., Nymick J. M., Shi M., Stolpen F. A., Ju J., Page Z. A. (2025). Multicolor Digital
Light Processing 3D Printing Enables Dissolvable Supports for Freestanding
and Non-Assembly Structures. ACS Central Science.

[2] Ponce I. A., Moran B., Hawker C. J., Shusteff M., Huang S. (2025). Dual-wavelength
simultaneous patterning of degradable thermoset supports for one-pot
embedded 3D printing. ACS Central Science.

[3] Zhang F., Zhu L., Li Z., Wang S., Shi J., Tang W., Li N., Yang J. (2021). The recent development of vat photopolymerization:
A review. Additive Manufacturing.

[4] Liu C., Guo X., Tong Y., Liu C., Mao K., Sun H., Hu Q., Gong H., Li X., Feng Y. (2024). Vat photopolymerization
3D printing SiBCN ceramic metamaterials with strong electromagnetic
wave absorption. Additive Manufacturing.

[5] Xu Y., Qi F., Mao H., Li S., Zhu Y., Gong J., Wang L., Malmstadt N., Chen Y. (2022). In-situ transfer vat
photopolymerization for transparent microfluidic device fabrication. Nat. Commun..

[6] Wu, J. ; Bai, C. ; Hu, D. ; Liu, D. ; Jiang, P. ; Wang, X. 10 - Vat photopolymerization 3D printing application in bioengineering. In Vat Photopolymerization Additive Manufacturing; Wang, X. , Ed.; Elsevier, 2024; pp 329–363.

[7] Jiang J., Xu X., Stringer J. (2018). Support Structures
for Additive Manufacturing: A Review. Journal
of Manufacturing and Materials Processing.

[8] Liang H., Hu B., Li R., Sun J., Wang Y., Zhou P., Cai P., Bai J. (2024). Enhancing mechanical integrity of vat photopolymerization
3D printed hydroxyapatite scaffolds through overcoming peeling defects. Journal of the European Ceramic Society.

[9] Nazir A., Gokcekaya O., Md Masum Billah K., Ertugrul O., Jiang J., Sun J., Hussain S. (2023). Multi-material additive manufacturing: A systematic
review of design, properties, applications, challenges, and 3D printing
of materials and cellular metamaterials. Materials
& Design.

[10] Guven E., Karpat Y., Cakmakci M. (2022). Improving
the dimensional accuracy
of micro parts 3D printed with projection-based continuous vat photopolymerization
using a model-based grayscale optimization method. Additive Manufacturing.

